# Use of Malachite Green-Loop Mediated Isothermal Amplification for Detection of *Plasmodium* spp. Parasites

**DOI:** 10.1371/journal.pone.0151437

**Published:** 2016-03-11

**Authors:** Naomi W. Lucchi, Dragan Ljolje, Luciana Silva-Flannery, Venkatachalam Udhayakumar

**Affiliations:** 1 Malaria Branch, Division of Parasitic Diseases and Malaria, Center for Global Health, Centers for Disease Control and Prevention, Atlanta, Georgia, United States of America; 2 Atlanta Research and Education Foundation/Veterans Affairs Medical center, Decatur Georgia, United States of America; Johns Hopkins Bloomberg School of Public Health, UNITED STATES

## Abstract

Malaria elimination efforts are hampered by the lack of sensitive tools to detect infections with low-level parasitemia, usually below the threshold of standard diagnostic methods, microscopy and rapid diagnostic tests. Isothermal nucleic acid amplification assays such as the loop-mediated isothermal amplification (LAMP), are well suited for field use as they do not require thermal cyclers to run the test. However, the use of specialized equipment, as described by many groups, reduces the versatility of the LAMP technique as a simple tool for use in endemic countries. In this study, the use of the malachite green (MG) dye, as a visual endpoint readout, together with a simple mini heat block was evaluated for the detection of malaria parasites. The assay was performed for 1 hour at 63°C and the results scored by 3 independent human readers. The limit of detection of the assay was determined using well-quantified *Plasmodium* spp. infected reference samples and its utility in testing clinical samples was determined using 190 pre-treatment specimens submitted for reference diagnosis of imported malaria in the United States. Use of a simplified boil and spin methods of DNA extraction from whole blood and filter paper was also investigated. We demonstrate the accurate and sensitive detection of malaria parasites using this assay with a detection limit ranging between 1–8 parasites/μL, supporting its applicability for the detection of infections with low parasite burden. This assay is compatible with the use of a simple boil and spin sample preparation method from both whole blood and filter papers without a loss of sensitivity. The MG-LAMP assay described here has great potential to extend the reach of molecular tools to settings where they are needed.

## Introduction

Molecular tests such as PCR-based assays are known to be more sensitive and specific in the detection of malaria parasites compared to microscopy and rapid diagnostic tests (RDTs). However, their use is limited by the fact that many molecular tools are technically challenging and require sophisticated equipment. Isothermal DNA amplification assays, such as the loop mediated isothermal amplification (LAMP) have been described [1]. These assays are well suited for field use in that they do not require a thermal cycler to run the test since the reaction is performed at a single temperature. Several studies have reported on the use of the LAMP assay for malaria parasite detection [2–10]. The LAMP assay results in the formation of magnesium pyrophosphates that are known to lead to an increase in turbidity as more DNA is amplified. This led to the initial LAMP readout in which the endpoint reaction was determined by the observable turbidity in the tube [11]. However, because of the subjectivity imbedded in this readout, further modifications to the LAMP assay have been made by employing automated equipment to read the endpoint. These have included the use of real-time turbidimeters [4, 6], real-time PCR machines [12], florescence real-time tube-scanners [5, 13, 14] and spectrophotometers [8] for LAMP product discrimination. The use of specialized equipment, however, reduces the versatility of the LAMP technique for poor and developing countries. Recently, a non-instrumented nucleic acid amplification by LAMP (NINA-LAMP) for malaria parasite detection was reported [7]. However, while this non-instrument LAMP assay has great potential for point-of-care and field use, one of its limitations is that it is not practical for large-scale population based assays since only 4–6 samples can be tested per run.

Colorimetric approaches for LAMP product readout have been investigated [8, 15] with some success. Recently, a malachite green (MG)-based LAMP assay was used for the detection of *Leishmania* species [16]. The MG dye was also successfully utilized, together with an isothermal amplification assay referred to as the genome exponential amplification reaction (GEAR) assay, for the detection of *Escherichia coli* O157:H7 [17]. MG is an organic compound that has traditionally been used as a dye for materials such as silk, leather, and paper. The MG-isothermal amplification assays (LAMP and GEAR) result in a green/blue color upon amplification of DNA while the negative samples (no amplification) are colorless [16, 17] and therefore no special automated readers are required. This visual assessment of LAMP and GEAR assay products using MG dye was shown to be reproducible, robust and consistent [16, 17]. The most attractive feature of the MG-LAMP assay is the fact that it can potentially be used for the screening of large number of samples since the assay can be performed using a heat block that maintains a constant temperature.

In this study, the use of the MG-LAMP together with a mini heat block for the detection of malaria parasites was evaluated to demonstrate the feasibility of using this tool for field based surveillance studies that might be employed in malaria elimination efforts.

## Materials and Methods

### Plasmodium parasites and clinical samples

The five human infecting Plasmodium species, *P*. *falciparum* (PHI, Santa Lucia, Nigeria 5, FC27 and Benin), *P*. *vivax* (Sal 1), *P*. *malariae* (Uganda I), *P*. *ovale* (Nigeria I) and *P*. *knowlesi* (H-strain) acquired from archived samples at the CDC, were used to determine the utility of the genus specific MG-malaria LAMP. The *P*. *falciparum* strains and the WHO international standard for *P*. *falciparum*, obtained from the National Institute for Biological Standards and control (NIBSC; Hertfordshire, UK) were used to determine the limits of detection of the MG-LAMP in this study. Clinical samples used in this study were obtained from a “Malaria drug resistance testing and surveillance” study that was reviewed and approved by the Centers for Disease Control and Prevention (CDC) Institutional Review Board. The study was deemed a routine surveillance activity and not a human subjects’ research activity and therefore consent from donors was waived by the institutional review board. The pre-treatment blood from patients presenting malaria symptoms after returning to the USA from visits to malaria endemic countries was submitted to the CDC by clinicians and/or state health departments for confirmatory malaria diagnosis.

### Malachite Green Malaria LAMP Method

**Fig 1:**
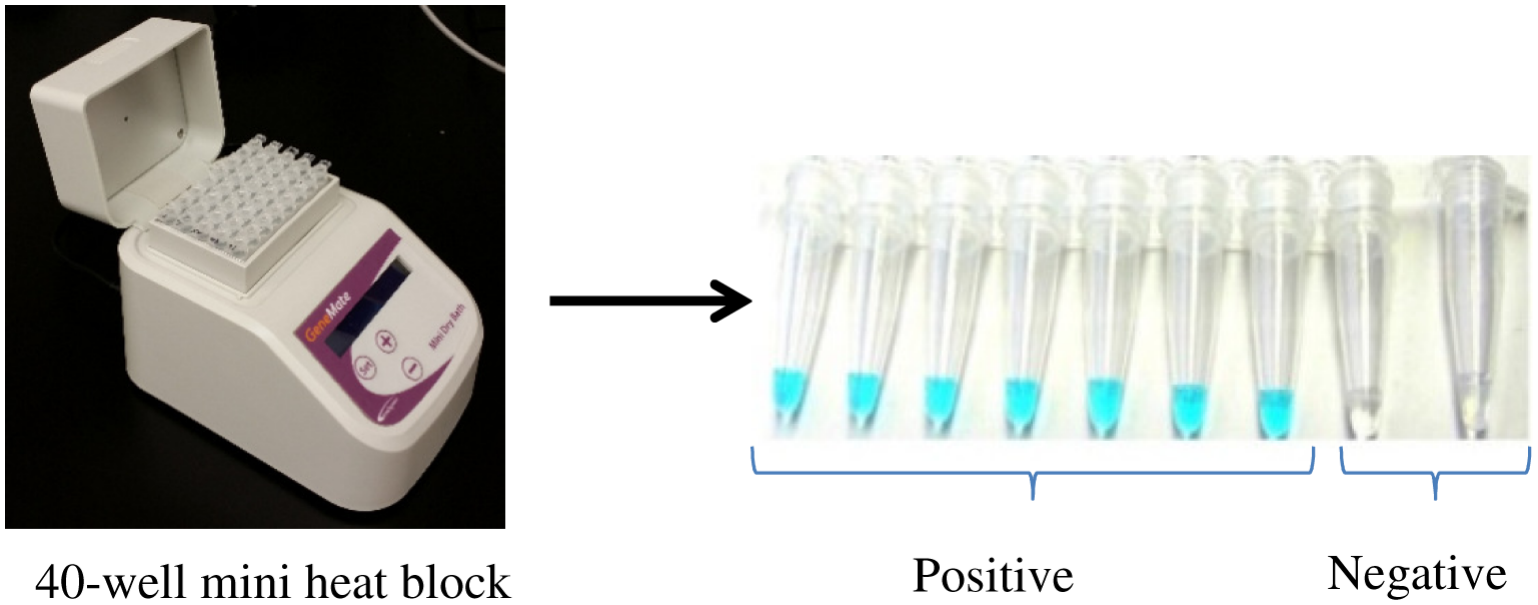
Expected results for the MG-LAMP assay.

The Malachite Green (MG) malaria LAMP (MG-LAMP) assay was performed in a 20 μL total reaction volume containing 2X in-house buffer (40 mM Tris-HCL pH 8.8, 20 mM KCl, 16 mM MgSO_4_, 20 mM (NH_4_)SO_4_, 0.2% Tween-20, 0.8 M Betaine, 2,8 mM of dNTPs each), 0.004% MG, 8 units of Bst polymerase (New England Biolabs, Ipswich, MA) and 5 μL of template DNA. Mitochondria *Plasmodium* genus-specific primers [6] were used to amplify the DNA. The amplification was carried out at 63°C for 60 minutes using a commercially available mini heat block (GeneMate, BioExpress, Utah, US). Samples were allowed to cool for 15 minutes before being scored by three independent human readers. Positive samples retained a light green/blue malachite green color while negative samples turned colorless, Fig 1.

### Determination of the optimum malachite green (MG) concentration

To determine the optimum concentration of MG to use in this LAMP assay, four different concentrations of MG were evaluated; 0.016%, 0.008%, 0.006% and 0.004%. Each concentration was tested in quadruplicate with a known *P*. *falciparum* (3D7) positive control and a no template control (NTC) consisting of DNase-free water. The reactions were tested three independent times and scored by 6 independent human readers. Each reader was requested to score “yes” if they observed a blue/green color and “no” if the tube was colorless. An indeterminate result was assigned when discordant results were obtained among the readers or when duplicate runs did not concur.

### QIAamp DNA Extraction

DNA was extracted from all clinical samples and quantified serial diluted *Plasmodium* samples using 200 μL of blood with the QIAamp blood kit (QIAGEN, Inc., Chatsworth, CA) according to the manufacturer’s instructions and stored at 4°C until processed.

### Boil and Spin method of DNA Extraction from Whole blood and Filter papers

A series of two-fold *P*. *falciparum* dilutions was used to investigate the use of a simpler sample preparation method using the boil and spin method, as previously described [5]. Briefly, 50μL of whole blood was boiled for 10 minutes at 99°C. The tubes were centrifuged for 5 minutes and 5μL of the supernatant used for the LAMP assay. FTA-micro elute cards were spotted with 40μL of whole blood, dried and stored with a desiccant at room temperature. DNA was extracted by punching out the complete circle of spotted blood. The punched pieces of filter papers were washed with 500μL of water and then boiled at 99°C for 10 minutes in 40μL of water. The tubes were centrifuged and 5μL of the supernatant used in the MG-LAMP assay.

### Determination of the limits of detection of the MG-LAMP assay

To determine the limits of detection (LoD) of the MG-LAMP assay, five sets of two-fold serially diluted *P*. *falciparum* isolates (PHI, Santa Lucia, Nigeria 5, FC27 and Benin) and the WHO standard for *P*. *falciparum* were used. In addition, three strains of *P*. *malariae*, *P*. *ovale*, and two of *P*. *vivax* were tested. Two-fold serial dilutions of each of these samples were carefully prepared using malaria—free whole blood starting with a concentration of 2,000 parasites/μL to 1 parasites/μL. The dilutions were tested two times each in triplicates or duplicates and scored by three independent human readers.

### Determination of the clinical sensitivity and specificity of the MG-LAMP assay

A total of 190 clinical samples {both malaria positive (101 *P*. *falciparum*, 29 *P*. *vivax*, 13 *P*. *ovale*, 6 *P*. *malariae* samples and 2 samples species not determined) and negative (39)} randomly selected from the pool of suspected imported malaria cases in the USA were used to evaluate the utility of the Plasmodium MG-LAMP assay for the detection of parasites in clinical samples. All the clinical samples were tested in duplicates and results were scored by three independent human readers. Results were compared to the real-time photo-induced electron transfer (PET)-PCR assay as previously described [17]. Parasite densities are not always available for the imported malaria cases which was why the real-time PCR was used. Briefly, the PET-PCR reaction was performed in a 20 μl reaction containing 2X TaqMan Environmental Master Mix 2.0 (Applied BioSystems), 250 nM each forward and reverse primer for *Plasmodium* genus and 2 μL of DNA template. The reactions were performed under the following cycling parameters: initial hot-start at 95°C for 15 minutes, followed by 45 cycles of denaturation at 95°C for 20 seconds, annealing at 63°C. Samples with a CT value of 40.0 or below were considered positive.

### Statistics

Sensitivity and specificity of MG-LAMP was calculated using the PET-PCR as the reference test. The percentage specificity and sensitivity were calculated using the formulae:

Sensitivity=truepositives/(truepositives+falsenegatives)×100

Specificity=truenegatives/(truenegatives+falsepositives)×100.

To assess the degree of agreement between human readers and the two diagnostic tests, kappa coefficients were calculated. The Probit analysis was used to determine the LoD for each *Plasmodium* species and for the crude DNA isolation methods. The LoDs were calculated using a linear logistic model that assesses the relationship between the probability of the response and the parasite concentration (parasites/μL). Data were entered into Microsoft Excel 2010 (Microsoft, Redmond, WA) and analysed using SPSS software, version 18 (SPSS Inc., Chicago, IL, USA). Precision of the estimates was determined by calculating exact 95% confidence intervals for each test statistic.

## Results

### Optimum MG concentration for use in the malaria MG-LAMP

A 100% concordance was observed among the 6 human readers using a final concentration of 0.004% of MG. Higher concentrations of MG resulted in an increase in false positivity, Table 1. The resulting blue/green color of the MG-LAMP assay was shown to remain intact for up to four weeks, the duration of time the tubes were kept at room temperature. Time points beyond 4 weeks was not investigated. Subsequent experiments were performed using a final concentration of 0.004% MG.

**Table 1:** Determination of the optimum MG concentration for use in the LAMP assay.

Final MG-Conc.		Color			
	Sample	Yes	No	% Positive	% Negative
**0.004%**	**Positive control**	72*	0	100	0
	**NTC**	0	72	0	100
**0.006%**	**Positive control**	72	0	100	0
	**NTC**	21	51	29.17	70.83
**0.008%**	**Positive control**	72	0	100	0
	**NTC**	20	51	27.78	70.83
**0.016%**	**Positive control**	72	0	100	0
	**NTC**	17	56	23.61	77.78

*Each concentration was tested three times in quadruplicates and scored by six human readers giving a total of 72 readings. NTC = no template control (negative control).

### Limits of detection of the malaria MG-LAMP

The malaria MG-LAMP assay was shown to detect the five human infecting *Plasmodium* species: *P*. *falciparum*, *P*. *vivax*, *P*. *malariae*, *P*. *ovale* and *P*. *knowlesi* with 100% concordance among three individual human readers. To determine the LoD of the assay, 2-fold serial dilutions of *P*. *falciparum (5 strains)*, *P*. *vivax (2 strains)*, *P*. *malariae (3 strains)* and *P*. *ovale (3 strains)* and the WHO standard (*P*. *falciparum*), were used. Each strain was tested three times and scored by three independent human readers.We observed 100% test positivity for parasite concentration between 2,000 to 4 parasites/μL for *P*. *falciparum*, *P*. *vivax* and *P*. *ovale* strains and 8 parasites/μL for *P*. *malariae*. The LoDs (parasites/μL) for each species as determined by Probit modelling yielded a LoD of 3 (95% CI: 2.361–4.467) for *P*. *falciparum*; 8 (95% CI: 5.33–16.296) for *P*. *malariae* and 3 (95% CI: 1.769–8.98) for *P*. *ovale*. This analysis could not be performed for *P*. *vivax* since we observed 100% test positivity for all the parasite dilutions.

### MG-LAMP can be utilized with crude sample preparation methods

A series of 2-fold *P*. *falciparum* dilutions ranging from 1000–1 parasite/μL was used to investigate the effect of using simpler sample preparation methods from whole blood (WB) and FTA micro-elute filter papers. The assays were performed four times (WB) and three times (FTA micro elute cards) and scored by three independent human readers. The boil and spin extraction method using whole blood resulted in a LoD of 3.1 parasites/μL (95% CI: 2.091–18.505) and 3.5 parasites/μL (95% CI: 2.298–19.421) using FTA micro-elute cards parasites/μL.

### Utility of the MG-LAMP assay in detecting malaria parasites in clinical samples

The utility of the malaria MG-LAMP assay for use in clinical samples was determined by testing 190 retrospective clinical samples. Results were compared to a *Plasmodium* genus-specific real-time PET-PCR assay. Of the 190 clinical samples tested, 38 samples were found to be negative and 149 positive by both MG-LAMP and PET-PCR. Three samples were indeterminate by MG-LAMP in that one of the two runs was not in concordance with the other one, Table 2. Two of these indeterminate samples were positive by PET-PCR assay with Ct values of 38.45 and 38.61. The other sample was negative by the PET-PCR. Using the 187 samples with a positive or negative results i.e. excluding the indeterminate results, we observed a 100% sensitivity (CI: 97%-100%) and specificity (CI: 86%-100%) between the MG-LAMP and PET-PCR assays.

**Table 2:** Comparison of MG-LAMP to PET-PCR.

# of samples	Run 1			Run 2			MG-overall result	PET-PCR	Ct (PET-PCR)
	Reader1	Reader2	Reader3	Reader1	Reader2	Reader3			
38	Neg	Neg	Neg	Neg	Neg	Neg	Neg	Neg	No Ct
149	Pos	Pos	Pos	Pos	Pos	Pos	Pos	Pos	16.6–38.15
2	Pos	Pos	Pos	Neg	Neg	Neg	ID	Pos	38.45, 38.61
1	Neg	Neg	Neg	Pos	Pos	Pos	ID	Neg	No Ct

ID = indeterminate

### Agreement of human readers

We observed 100% agreement (Kappa = 1) among the human readers in all the experiments performed.

## Discussion

In this study, we demonstrate the accurate and sensitive detection of malaria parasites using a *Plasmodium* genus-specific MG-LAMP assay. This malaria MG-LAMP assay consistently detected samples with as low as 1 parasites/μL for *P*. *vivax*, 4 parasites/μL for *P*. *falciparum* and *P*. *ovale* and 8 parasites/μL for *P*. *malariae* supporting its applicability for the detection of infections with low parasite burden. Importantly, this assay was shown to be compatible with the use of a simple mini heat block and the simple boil and spin sample preparation method from both whole blood and filter papers without a loss of sensitivity implying that it can be used in low resource settings without the need of expensive equipment and tedious DNA extraction procedures. We calculated the cost of the MG-LAMP assay to be about $3.00 per test/sample if performed using an in-house 2x LAMP buffer and $5.05 using a commercial buffer. This cost does not include the DNA isolation step and the initial cost of the 40-well heat block which is approximately $489 (GeneMate BioExpress listed price). The MG-LAMP assay described here has great potential to extend the reach of molecular tools to settings where they are needed.

Of the 190 clinical samples tested, only three resulted in discordant results between the MG-LAMP and the real-time PET-PCR assay. Two of these samples were PET-PCR positive but MG-LAMP indeterminate; defined as positive in one run but negative in the replicate run. Both of these samples had particularly high Ct values (38.45 and 38.61) obtained by the PET-PCR. These PET-PCR Ct values correspond to a parasite density of approximately 2 parasites/μL [18]. The other indeterminate sample was found to be negative by the PET-PCR. It is likely that this sample is also of very low parasite density, not detectable by PET-PCR and “partially” by the MG-LAMP assay. Due to limitations in the amount of sample available for testing, we were unable to perform additional runs with these clinical samples. Samples with low parasite densities present an interesting situation as their consistent detection depends on the limits of detection of the assays utilized to test them and are more prone to stochastic noise. Inconsistencies in results obtained when testing samples with low parasite densities or at the limit of detection of the testing assay have been observed even when using the same molecular protocol [19, 20] Therefore, taking these details into consideration, we conclude that the malaria MG-LAMP assay and the PET-PCR assay are comparable in their detection limits and sensitivity.

The advantages and utility of the LAMP technique for use in endemic countries is now well acknowledged [21, 22] however, the use of specialized automated equipment such as turbidimeters and fluorescence readers reduces the versatility of the LAMP technique for use in large-scale surveys and field settings. Colorimetric approaches for LAMP product readout can help overcome this limitation. However, these need to provide a clear, non-subjective result. In all the experiments that were performed in this study, 100% concordance was observed among all the individual human readers that scored the results in the investigations described here. This finding supports the notion that this colorimetric assay can be confidently utilized in LAMP assays. As a note of caution, our results indicate that MG concentrations higher than 0.004% can lead to false positive results. This can be a limitation in settings where precise measurement are unreliable.

The malaria MG-LAMP assay developed here has some advantages that make it a viable alternative for use in malaria endemic regions for large scale population based surveys: 1) the MG signal is highly sensitive and non-subjective, facilitating the visual discrimination of results without any specialized and costly equipment such as turbidimeters or fluorescence readers, 2) a large number of samples (38 in our case) can be tested at the same time and if need be, additional heat blocks can be included if the testing of large number of samples is required, 3) it is easily accomplished in a closed system by the addition of the MG in the LAMP reaction mixture prior to amplification and therefore it does not require any post-amplification manipulation, often associated with contamination, 4) the fact that the developed color was seen to last for up to 4 weeks without fading implies that the tubes can be stored for recording purposes and follow-up quality control purposes and 5) the MG dye does not require any special storage conditions as it can be kept at room temperature without loss of activity.

The MG dye has previously been used in isothermal application including LAMP for *Leishmania* detection [16] and in GEAR assay for the detection of *Escherichia coli* O157:H7 [17]. Both studies reported the visual assessment of amplification produce using the MG dye as reproducible, robust and consistent. These studies together with the study described here demonstrate that the use of MG dye as a read out for LAMP assays provides a user-friendly colorimetric detection assay suitable for different infective agents that can be used for a high-throughput tool for the assessment of infections under field conditions in endemic countries.

The success of any malaria control program is measured by the reduction of malaria cases and of transmission. In order to determine this, it is important that all existing malaria cases including sub-microscopic or sub-RDT are detected. The MG-LAMP assay described here has great potential to extend the reach of molecular tools to settings where they are needed.
